# Chemokine CXCL12 and its receptor CXCR4 expression are associated with perineural invasion of prostate cancer

**DOI:** 10.1186/1756-9966-27-62

**Published:** 2008-11-04

**Authors:** Shiwu Zhang, Lisha Qi, Man Li, Danfang Zhang, Shaoyan Xu, Ning Wang, Baocun Sun

**Affiliations:** 1 Department of Pathology, Tianjin Cancer Hospital, Tianjin Medical University, Tianjin 300060, PR China; 2 Department of Pathology, Tianjin Dongli Hospital, Dongli District, Tianjin, 300300, PR China; 3 Department of Digestion, the Second Hospital, Tianjin Medical University, Tianjin 300211, PR China; 4 Department of Pathology, Tianjin Medical University, Tianjin 300070, PR China

## Abstract

**Objective:**

To identify the roles of CXCL12 and CXCR4 and the associated mechanism involved in perineural invasion of prostate cancer.

**Methods:**

The distribution and expression of CXCL12, CXCR4, MMP-2 and MMP-9 in human prostate cancer and in tumor cells invading nerve tissue were studied with immunohistochemical staining. The effects of exogenous CXCL12 and CXCR4 antagonist AMD3100 on PC3 prostate cancer cells invasiveness were assessed in vitro and in vivo.

**Results:**

The expression of CXCL12, CXCR4, MMP-2, and MMP-9 in human prostate cancer were higher than those in hyperplastic prostate tissues (*P *< 0.05). In vitro CXCL12 could stimulate the PC3 cells invasiveness (*P *< 0.05) while AMD3100 could inhibit invasiveness. In vivo, the number of nerves around the tumor tissue in the group treated with CXCL12 was significantly higher than that found in the control group (*P *< 0.05). Both the control group and the CXCL12-treated group had more nerves number near the tumor tissue than it found in the AMD3100-treated group. The positive cell number of CXCL12, CXCR4, MMP-2, MMP-9, and NGF expression ranked from highest to lowest, were the CXCL12-treated, the control, and the AMD3100-treated group(*P *< 0.05).

**Conclusion:**

CXCL12 and its receptor CXCR4 along with MMP-2 and MMP-9 are related with prostate cancer perineural invasion.

## Introduction

Perineural invasion (PNI) of malignant tumor cells means tumor cells tend to encroach upon nerve fibers and invade the lamellar sheath, leading to infiltration and metastasis. This phenomenon is often seen in adenoid cystic carcinoma, pancreatic cancer and prostate cancer [1]. Prostate cancer PNI usually localizes in a few important nerves, influencing the patients' quality of life and making it difficult to remedy. Residual tumor cells in nerve tissue may contribute to local recurrence and metastasis and influence prognosis [2]. Other studies have shown that the mechanism of PNI involves the microenvironment around the nerve tract that is suitable for tumor cell growth and proliferation. This situation promotes tumor cell infiltration and diffusion along nerve fibers [3].

Chemokines are small, secreted peptides that control adhesion and transendothelial migration of leukocytes, lymphocytes, and monocytes, especially during immune and inflammatory reactions. Chemokines combine with G-protein-coupled-receptors to perform their function. Chemokines and their receptors have significant roles in tumor metastasis, recurrence and angiogenesis [4]. Among the family of chemokines and their receptors that significantly contribute to the initiation and development of tumors, CXCL12 and its receptor CXCR4 are the most frequently mentioned. CXCR4 is the unique receptor for CXCL12, also named stromal cell-derived factor 1 (SDF1). Prostate cancer cells secrete many chemokines and their receptors, also found in cells in other tissues of the body, and these chemokines are involved in the prostate cancer cells' organ-specific metastasis [5]. During the process of prostate cancer PNI, MMP-2 and MMP-9 play an important role in degrading the matrix around the tumor and the nerve tissue. Our research aims to study CXCL12, CXCR4, MMP-2 and MMP-9 expression in prostate cancer PNI using in vitro experiments, animal model experiments and human prostate cancer tissue. The study was the first time to report the roles of CXCL12 and CXCR4 in PNI of prostate cancer.

## Materials and methods

### Cell lines and materials

Human androgen-independent prostate cancer cell line PC3 was provided by the central experiment lab of Tianjin Cancer Hospital.

### Transwell experiment

PC3 cells were cultured in RPMI 1640 without serum at 37°C with 5% CO_2 _and were adjusted to 1 × 10^5^-1 × 10^6 ^cells/ml. The liquid extracellular matrix (ECM) gel (Sigma-Aldrich, St. Louis, USA) was pre-cooled to 2–8°C and 16.5μl was added to the upper surface of each Transwell chamber filter (Corning corporation, New York, USA). The chambers were incubated at 37°C with 5% CO_2 _for one hour to allow the matrix to solidify and then 1 × 10^4^-1 × 10^5 ^PC3 cells in suspension were added to the upper chambers. For the controls, 600 μl serum-free medium was added to the lower chamber. For the CXCL12 experimental wells, 600 μl serum-free medium supplemented with 0.1 μg CXCL12 (Jingmei corporation, Tianjin, China, reconstituted with Saline) was added to the lower chamber. For the CXCR4 antagonist experimental wells, 600 μl serum-free media supplemented with 12 pmol AMD3100 (Sigma corporation, USA, reconstituted with Saline) was added to the lower chamber. The chambers were incubated for 20 hours at 37°C with 5% CO_2_. The upper surface of the filter was removed using a cotton swab. Cells that invaded through the ECM and the filter to the lower surface were fixed using 5% glutaraldehyde and were then stained with H&E. The cell number in five fields (up, down, median, left, right. ×400) were counted for each chamber, and the average was determined. Each experimental condition was repeated four times.

### Model of prostate cancer and tumor experiments in BALB/C nude mice

BALB/C nude mice were obtained from the Academy of Military Medical Sciences of China. Six to eight-week-old, 20 to 25 g BALB/C nude mice (14 males and 14 females) were used to establish the perineural invasion model. Each mouse received a groin injection of 1 × 10^6 ^PC3 cells. On the 18th day after inoculation, the mice were divided randomly into three groups and received treatment for 10 days. In the CXCL12 group (n = 8) each mouse was treated with CXCL12 at 0.1 μg/day[6]. In the AMD3100 group (n = 10) each mouse was treated with 10 pmol/day AMD3100[7]. In the control group (n = 10) each mouse was treated with 0.1 ml/day sodium chloride. The mice were sacrificed after 10 days of treatment, and the tumor mass and surrounding tissue were removed, fixed with formalin and made into tissue sections.

### Counting the number of nerves around the tumor tissue in H&E stained sections

Microscopic analysis was performed on H&E stained sections. The nerves within one field (400×) of the tumor tissue were defined as being near the tumor. The nerves away from the tumor tissue more than one field (400×) were considered far from the tumor. The average number of nerves near and far from the tumor tissue were determined by counting the mean value in five fields (400×).

### Immunohistochemical staining and quantification

Polyclonal antibodies used in this study were rabbit anti-MMP-2 (Boster Wuhan, China, dilution 1:100), rabbit anti-MMP-9 (Boster Wuhan, China, dilution 1:100), rabbit anti-CXCL12 (Boster Wuhan, China, dilution 1:150), rabbit anti-CXCR4 (Boster Wuhan, China, dilution 1:100) and rabbit anti-NGF (Boster Wuhan, China, dilution 1:100). Four-micrometer sections of formalin-fixed paraffin-embedded tissue were mounted on poly-L-lysine-coated slides. The slides were air-dried and the tissue was deparaffinized. Endogenous peroxidase activity was blocked with 3% hydrogen peroxide in 50% methanol for 10 min at room temperature. The sections were rehydrated and washed with PBS and then pretreated with citrate buffer (0.01 M citric acid, pH 6.0) for 20 min at 100°C in a microwave oven. After nonspecific binding sites were blocked by incubating in 2% normal goat serum in phosphate-buffered saline (PBS) for 15 min at 37°C, the sections were incubated overnight at 4°C with the primary antibody. The sections were then washed with PBS and incubated with the secondary HRP-conjugated antibody for 30 min at 37°C. Staining was performed by incubating with fresh 3,3'-diaminobenzidine (DAB) buffer. The sections were washed in running water and counterstained with hematoxylin, followed by dehydration and mounting. PBS was utilized in place of the first antibody for the negative control.

When stained for MMP-2, MMP-9, CXCL12, CXCR4 and NGF, tumor cells with brown cytoplasm were considered positive. We observed 10 fields per section at 400× magnification, and positive cell numbers were counted in 100 random melanoma cells in every field. The mean percentage of positive cells was used to determine the expression of the proteins in a section. All these counts were blindly performed in at least 3 randomly chosen sections from each mouse.

### Clinical data and pathological material for human prostate cancer cases

Clinical data and pathological material were collected for 22 prostate cancer patients including ten cases of Gleason grade IV and twelve cases of Gleason grade V and 20 patients with prostate hyperplasia who were seen in the Tianjin Cancer Hospital between 1985 and 2005. The median age of cancer patients was 68.40 ± 9.55 years (range 57–85 years). The detail clinical and pathological characteristics of 42 cases of samples were listed in a table 1. All these cancer patients were treated with surgery and the surgical margin were negative.

**Table 1:** The clinical and pathological characteristics of 42 cases of prostate cancer and prostate hyperplasia

	group	clinical and pathological characteristics
Mean age (range)	Prostate cancer	68.40 ± 9.55 (57–85)
	prostate hyperplasia	64.18 ± 11.74 (45–81)
Histologic examination	Prostate cancer	adenocarcinoma
	prostate hyperplasia	Fibromuscuadenomaoid node, adenomaoid node, leiomyomaoid node and fibrohemangioid
Pathological evaluation	Prostate cancer	Gleason grade IV 10(45.5%) and Gleason grade V 12(54.5%)
	prostate hyperplasia	Moderate hyperplasia 12(60%) and severe hyperplasia 8(40%)
Volume(cm^3^)	Prostate cancer	78.53 ± 29.75
	prostate hyperplasia	45.25 ± 18.97
Local invasion and metastasis	Prostate cancer	Local invasion 14(63.4) and metastasis 2(9%)
	prostate hyperplasia	no
Level of prostate specific antigen in blood(ng/ml)	Prostate cancer	68.57 ± 50.43
	prostate hyperplasia	20.21 ± 3.27

### Statistical analysis

Statistical software SPSS 10.0 (Chicago, Illinois) was used to perform the analysis. Differences among groups were assessed using the ANOVA test, and the least significant difference (LSD) test was used to compare the differences between every combination of two groups. Differences between two groups were assessed using a *t *test. A P value less than 0.05 was considered statistically significant.

## Results

### The invasiveness of human prostate cancer PC3 cells

**Figure 1:**
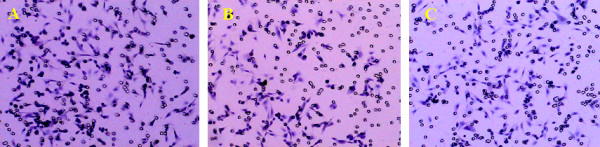
**A: CXCL12-treated group.** The number of invading PC3 cells was higher than that found in the other two groups. H&E, 100×. B: Untreated control group. Some PC3 cells migrated through the ECM gel to the lower layer. H&E, 100×. C: AMD3100-treated group. The number of invading PC3 cells was much lower than that found in the other two groups. H&E, 100×.

In vitro, the invasiveness of PC3 cells treated with CXCL12 increased greatly. The average number of cells invading through the Transwell chamber filter was 169.7(Figure 1A). The invasiveness was inhibited in the cells treated with the CXCR4-specific inhibitor AMD3100 (Figure 1B). The average number of cells invading through the Transwell chamber filter was 77.3, lower than the 115.45 in the untreated control group (Figure 1C). The differences among the three groups were statistically significant (*F *= 32.00, *P *< 0.05) (table 2).

**Table 2:** The average number of invading cells in the three groups

Groups	CXCL12-treated group (n = 4)	Untreated control group (n = 4)	AMD3100-treated group (n = 4)
Average number of invading cells	169.7 ± 47.23#	115.45 ± 26.49	77.30 ± 33.30
*F *value		32.00	

# The differences of average number of invading cells between the CXCL12-treated group and the control group is statistically significant. (*P *< 0.05).

### Tumor growth in inoculated animals

**Figure 2:**
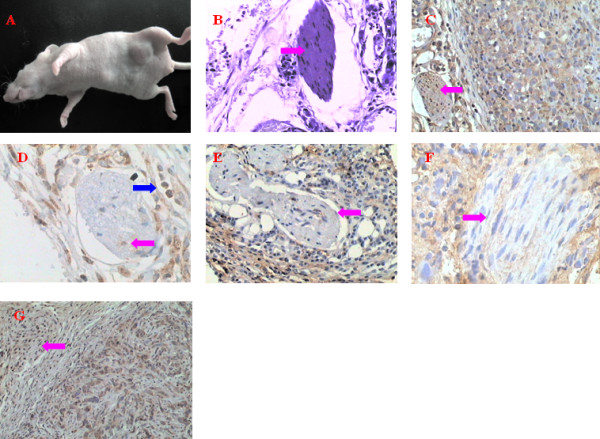
**A: The gross appearance of tumor mass present in a BALB/C mouse 28 days after inoculation with PC3 prostate cancer cells.** B: Sporadic PC3 cells infiltrate into nerve tissue. The arrows indicate nerve tissue. H&E, 100×. C: Sporadic PC3 cells infiltrated the nerve. Both PC3 cells and nerve tissue express NGF. The red arrows indicated nerve tissue. NGF immunohistochemical staining (SP), 100×. D: Sporadic PC3 cells infiltrate the nerve. Both PC3 cells and nerve tissue expressed CXCL12. Red arrow indicates nerve tissue secreting CXCL12. Blue arrow indicates tumor cells. CXCL12 immunohistochemical staining (SP), 100×. E: Sporadic PC3 cells infiltrate the nerve. PC3 cells expresse CXCR4. The arrow indicate nerve tissue. CXCR4 immunohistochemical staining (SP), 100×. F: PC3 cells express MMP-2 that degrades ECM around nerve tissue and promotes PNI. The red arrow indicates nerve tissue. MMP-2 immunohistochemical staining (SP), 200×. G: PC3 cells express MMP-9 that degrades ECM around nerve tissue and promotes PNI. The red arrow indicates the nerve tissue. MMP-9 immunohistochemical staining (SP), 200×.

Tumor mass could be palpabled in the groin of the BALB/C mice 10 days after inoculation. Treatments were administrated for 10 days starting on the 19^th ^day after inoculation. The average diameter of tumor was about 0.8 cm when the mice were sacrificed (Figure 2A). There were no deaths throughout the duration of the experiment until the mice were sacrificed.

### Pathological examination

The tumors appeared to be nodular, and most of them had complete pseudo-encapsulation. The tumor cross section was light pink or pale. Histologically, PC3 cells had moderate eosinophilic cytoplasm, round nuclei and prominent nucleoli. Many nerves grew around the tumor lesion. Some tumor cells surrounded nerves and some tumor cells invaded into the lamellar sheath (Figure 2B).

The average number of nerves near the tumor tissue in the AMD3100-treated group was lowest, and the CXCL12-treated group had the highest number of nerves near the tumor tissue. The average number of nerves near the tumor was statistically higher than the average number of nerves far from the tumor in both the CXCL12-treated group and the control group (F = 9.49, *P *< 0.05). Tumors in the AMD3100-treated group seemed to have more nerves near the tumor than far from the tumor (table 3).

**Table 3:** The average number of nerves near and far from the tumor tissue in the three groups

Groups	CXCL12-treated group (n = 8)	Untreated control group (n = 10)	AMD3100-treated group (n = 10)
Number of nerves near tumor	8.73 ± 4.42*#	5.11 ± 2.39*	2.80 ± 1.19#
Number of nerves far from tumor	1.91 ± 1.10	2.22 ± 1.64	1.24 ± 0.66

*F *value	9.49		

# The differences of the number of nerves between the CXCL12-treated group and the control group and between the AMD3100-treated group and the control group are statistically significant. (*P *< 0.05).* The differences between the number of nerves near the tumor and the number of nerves far from the tumor in CXCL12-treated group and untreated control group are statistically significant (*P *< 0.05).

### The detection of CXCL12, CXCR4, MMP-2, MMP-9, and NGF expression by immunohistochemical staining

NGF was highly expressed in tumor cells as well as nerve tissue around the tumor (Figure 2C). Among the three groups, all of these proteins were expressed most strongly in the tumors of the CXCL12-treated group and most weakly in the AMD3100-treated group. CXCL12 expression was detected in tumor cells and Schwann cells around the tumor by immunohistochemical staining, and some weak staining was found occasionally in mesenchymal cells (Figure 2D). CXCR4 was strongly expressed in tumor cells (Figure 2E). Clear immunohistochemical staining for MMP-2 and MMP-9 was detected in tumor cells and some mesenchymal cells (Figures 2F &2G). The positive cell number of CXCL12, CXCR4, MMP-2, MMP-9 and NGF expression of tumor cells in CXCL12-treated group was the highest. The differences in the number of positive cells for these proteins among the three groups were statistically significant (*F *= 18.77 for CXCL12, 78.05 for CXCR4, 40.54 for MMP-2, 32.66 for MMP-9, 22.54 for NGF, *P *< 0.05) (table 4).

**Table 4:** The average positive cell number of CXCL12, CXCR4, MMP-2, MMP-9, and NGF expression in the three groups ($\bar{x}$ ± s)

Treatment	n	CXCL12	CXCR4	MMP-2	MMP-9	NGF
AMD3100	10	11.64 ± 4.96	19.81 ± 0.91	13.94 ± 4.63	12.96 ± 3.20	24.25 ± 5.41
Untreated control	10	18.30 ± 6.53	26.09 ± 5.48	22.47 ± 3.30	18.72 ± 3.26	31.72 ± 3.38
CXCL12	8	34.63 ± 5.04	53.83 ± 5.75	41.03 ± 8.21	32.97 ± 6.97	51.24 ± 13.87

*F *value		18.77	78.05	40.54	32.66	22.54

The differences of positive cell number of CXCL12, CXCR4, MMP-2, MMP-9 and NGF expression of tumor cells among the three groups were all statistically significant(*P *< 0.05).

### The expression of CXCL12, CXCR4, MMP-2 and MMP-9 in human prostate tissue

**Figure 3:**
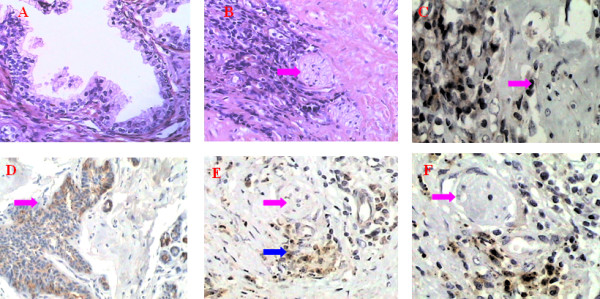
**A: The cells in hyperplastic prostate tissue differentiated well with glandular tube-like arrangement.** H&E, 100×. B: Human prostate cancer tumor cells grow around the nerve. Some tumor cells have invaded into nerve tissue. The red arrow indicates the nerve, H&E, 100×. C: Cells in a human prostate cancer tumor around the nerve express MMP-2 that degrades the ECM around the nerve tissue and promotes PNI. The red arrow indicates nerve tissue. MMP-2 immunohistochemical staining (SP), 100×. D: Cells in human prostate cancer tumor around the nerve express MMP-9 that degrades the ECM around the nerve tissue and promotes PNI. The red arrow indicates nerve tissue. MMP-9 immunohistochemical staining (SP), 100×. E: Prostate cancer cells infiltrate the nerve. Both tumor cells and nerve tissue express CXCL12. Red arrow indicates nerve. Blue arrow indicates tumor cells. CXCL12 immunohistochemical staining (SP), 100×. F: Prostate cancer cells expressing CXCR4 infiltrate the nerve. The red arrow indicates nerve tissue. CXCR4 immunohistochemical staining (SP), 100×.

The cells in hyperplastic prostate tissue differentiated well with glandular tube-like arrangement (Figure 3A). In prostate cancer tissue, many nerves grew around the tumor tissue. Some tumor cells surrounded nerves and invaded into the lamellar sheath (Figure 3B). Almost all tumor cells were positive for MMP-2 and MMP-9 staining (Figure 3C–D). The expression of these two proteins in prostate cancer was statistically higher than in prostate hyperplasia (t = 3.55; t = 2.38; *P *< 0.05) PNI was common in human prostate cancer. Results of immunohistochemical staining showed that both nerves and tumor cells expressed CXCL12 (Figure 3E). CXCL12 expression in prostate cancer was significantly stronger than in prostate hyperplasia (t = 4.55, *P *< 0.05). CXCR4 was expressed mainly in tumor cells, and it was also detected, but less intensely, in prostate hyperplasia (t = 3.86, *P *< 0.05) (Figure 3F) (table 5).

**Table 5:** The average positive cell number of CXCL12, CXCR4, MMP-2, and MMP-9 expression in prostate cancer and prostate hyperplasia ($\bar{x}$ ± s)

groups	n	CXCL12	CXCR4	MMP-2	MMP-9
Prostate cancer	22	37.30 ± 13.24*	37.09 ± 14.66*	42.86 ± 15.44*	52.48 ± 23.51*
Prostate hyperplasia	20	2.87 ± 1.17	3.27 ± 0.71	11.23 ± 5.10	19.22 ± 4.75

t value		4.55	3.86	3.55	2.38

*The difference between prostate cancer and prostate hyperplasia is statistically significant (*P *< 0.05).

## Discussion

Prostate cancer is the most commonly diagnosed malignancy and the second leading cause of cancer-related deaths in the Western male population[8]. Its incidence in China has greatly increased from 1.71 per 100,000 men in 1993 to 7.9 per 100,000 men in 2005. PNI is one of the most important characteristic of prostate cancer, as well as the cause of frequent recurrence and poor prognosis. PNI usually occurs in critical local nerves, and influences patients' quality of life and causes the disease difficult to cure[9]. Recurrence and metastasis are common when the disease is diagnosed in the later stages and mean a poor prognosis. Residual tumor tissue in nerve tissue is to blame for extracapsular spread and recurrence, which often results in treatment failure. Reports suggest that 79% of prostate cancer samples obtained during surgery display PNI [10] and that PNI is associated with tumor pathological grade, clinical stage and Gleason score. Elucidation of the trigger and mechanism involved in prostate cancer PNI is critical for improving prognosis and promoting clinicians choose the most appropriate therapy.

Besides their well-established roles in the inflammatory reaction, chemokines participate in many physiological and pathological behaviors including lymphocyte homing, immunity, infection, autoimmune disease, angiogenesis and so on [11]. Of late, increasing evidences suggested that chemokines were closely related to tumor growth, invasion and metastasis[12]. Among the family of chemokines and their receptors that significantly contribute to the initiation, progression, invasion and metastasis of tumors, tumor cells expressing high CXCR4 are often found in tissue expressing CXCL12, perhaps due to organ-specific metastasis[13]. One possible mechanism involves chemokines combining with their receptors to induce membrane wrinkling in tumor cells that forms pseudopodia. The tumor cells then adhere to and traverse through the extracellular matrix and basal membrane to enter the blood circulation and metastasize. However, there are not reports about chemokines and their recepters involving with PNI. Furthermore, The previous views suggested that tumor cells express high CXCR4 and the metastasis-targeted organs express high CXCL12, so the tumor cells were attracted to the ligand in these organs[14]. In our study, tumor cells not only expressed CXCR4, but also CXCL12. CXCR4 and CXCL12 jointly promote prostate cancer PNI. This phenomenon indicates that there may be autocrine stimulation by CXCL12 in tumor tissue. Some research validated that tumor cells can also express CXCL12. Taichman[15] showed that PC3 and DU145 prostate cancer cell lines and hormone-resistant LnCaP and C4-2B prostate cancer cell lines can express CXCL12. Prostate cancer cells with significant CXCR4 expression may escape from primary tumor foci, enter into the lymphatic and blood vessels and migrate toward cells expressing CXCL12. CXCL12 in normal tissue attracts the CXCR4 on the cancer cells, stimulating cell proliferation and inducing angiogenesis [16-18].

The previous explanation for tumor PNI is that interstitial tension around nerves is generally weak, making the microenvironment surrounding the lamellar sheath suitable for tumor cell growth. However, this explanation cannot adequately detail the molecular mechanism involved in prostate cancer PNI. Our study concludes with the idea that chemokines and their receptors interacting with MMPs contributed to prostate cancer PNI. CXCL12 can induce tumor cell secretion of MMP-9 that then degrades the blood vessel basal membrane, remodels vessels and stimulates growth of new vessels. All of these features together make it possible for tumor cells to easily enter the circulation. At the same time, MMPs can similarly induce CXCR4 expression in tumor cells.

Perissinotto[19] reported that CXCL12 can markedly upregulate the expression and activity of MMP-9 in osteosarcoma. In the progression of prostate cancer PNI, CXCL12 and CXCR4 secreted by tumor cells and nerve tissue induce tumor cells to migrate toward nerves. At the same time, tumor cells secrete MMP-2 and MMP-9 that degrade the matrix around the tumor and the nerve tissue, promoting PNI. Our research demonstrated that the expression of CXCL12, CXCR4, MMP-2, and MMP-9 in prostate cancer was much higher than in hyperplastic prostate tissue, implying that these proteins interact with each other and together may regulate chemotactic ability and invasiveness of tumor cells.

Exogenous CXCL12 can enhance PC3 cells' ability to penetrate ECM gel. Initially in this process, chemokines combining with their receptors cause actin to concentrate and enhance cell mobility. Muller [20] reported that CXCL12 could increase the concentration of F-actin in breast cancer cells in vivo, thus facilitate the breast cancer cells' migration and invasion in a specific direction. Secondly, CXCR4 combining with CXC12 can rapidly stimulate transmembrane transporting of Ca^2+^. Vaday[21] showed the specific antibody against human CXCR4 scFv could significantly inhibit the Ca^2+ ^transport mediated by CXCL12 and CXCR4. Thirdly, CXCL12 promotes tumor cell adhesion to the basement membrane components.

AMD3100 is a small highly specific non-peptide antagonist of CXCR4[6]. It can inhibit the signal transduction of CXCL12 mainly through a route that does not involve the ligand-receptor interaction. AMD3100 is capable of inhibiting infiltration by acute lymphoblastic leukemia and ovarian cancer cells[7]. AMD3100 competitively inhibited the CXCR4 binding with CXCL12 and blocked the downstream pathway. In addition, AMD3100 can promote MMP secretion by blocking the downstream of signal transduction pathway about the CXCL12 and CXCR4 interaction.

Nerve growth factor (NGF) not only promotes nerve tissue growth, development and regeneration, but regulates the growth and metastasis of pancreatic cancer, prostate cancer, lung cancer and retinal glioblastoma cells[22]. Results of Oelmann et al[23]. indicated NGF promotes proliferation in three kinds of lung cancer cell lines, and the effect took on dose-dependent. This effect could be blocked by NGF antibody. Research from Zhu et al. [24] showed that pancreatic cancer cells expressed NGF and lamellar sheath expressed TrkA, a combination of receptor and ligand that attracts tumor cells to nerves, leading to invasion and metastasis along nerves. In addition, NGF stimulated the expression of MMPs in tumor cells. Our results indicated that the expression of NGF in the CXCL12-treated group was much higher than that in the control group and the AMD3100-treated group. Of the three groups, the CXCL12-treated group had the highest average number of nerves around tumor. This study suggested that CXCL12 could increase NGF expression in PC3 cells. NGF could combine with its receptor expressed on the cell surface to activate the corresponding signal transduction pathway, enhancing the cells' invasiveness and mobility toward nerves. NGF secreted by tumor cells could also enhance the synthesis and release of some factors including MMPs that promoted cell invasion and metastasis. Furthermore, NGF diffuses around tumor tissue and stimulates nerve growth along this concentration gradient, enabling tumor cells to invade nerves and metastasize. Meanwhile, the increased number of nerves in tumor mass can secrete numerous factors to promote tumor cell growth.

## Abbreviations

PNI: perineural invasion; CXCL12: C-X-C chemokine ligand 12; CXCR4: C-X-C chemokine receptor 4; rpm: revolutions per minute; PBS: phosphate-buffered saline; MMP: matrix metalloproteinase; NGF: nerve growth factor; VEGF: vascular endothelial growth factor

## Competing interests

The authors declare that they have no competing interests.

## Authors' contributions

ZS carried out the animal experiment and cell culture. QL carried out the experiment in vitro. LM carried out morphological observation and participated in the data collection. ZD carried out the immunohistochemical staining and counting. XS performed the statistical analysis. WN participated in the animal experiment and coordination. SB carried out the design of the study and helped to draft the manuscript. All authors read and approved the final manuscript.
